# Assessment of various salt water stresses with mineralization on plant growth and antioxidants activity to regulate oxidative stress and ROS scavenging of halophyte ice plant

**DOI:** 10.3389/fpls.2025.1626676

**Published:** 2025-09-15

**Authors:** Xinchao Ma, Shaoliang Zhou, Xuefei Guo, Lang Xin, Yao Guan

**Affiliations:** ^1^ Tarim University, Alar, China; ^2^ Xinjiang Production & Construction Corps Key Laboratory of Facility, Alar, China; ^3^ Yin Feihu Academician Workstation, Alar, China

**Keywords:** ice plant, antioxidant activity, hydrogen peroxide, halophyte, peroxidase, salinity stress

## Abstract

Halophyte such as ice plant is subjected to salinity and drought stress in their natural habitats, but our understanding of the effects of combined stress on plants is limited. This study aimed to investigate the mechanisms by which ice plant responds to combined salinity and alkalinity stress, which are currently unknown. This study evaluated the combined effects of salinity and alkalinity stress levels on the growth, mineral content, and phytochemical composition of *Mesembryanthemum crystallinum*. Therefore, experiment consisted of seven different salt treatments CK: distilled water control; T1: saline-alkali solution of 5 g/L; T2: saline-alkali solution of 10 g/L; T3: saline-alkali solution of 15 g/L; T4: saline-alkali solution of 20 g/L; T5: saline-alkali solution of 25 g/L; T6: saline-alkali solution of 30 g/L. The specific configuration method of the composite saline-alkali solution is to mix Na_2_SO_4_, NaCl, NaHCO_3_, CaCl_2_, MgCl_2_ in the proportion of 8:8:1:1:1 on the basis of distilled water. The results indicated that the T6 treatment (saline-alkali solution of 30 g/L) strongly decreased the plant height, stem thickness, leaf area and SPAD value of ice plants, and obviously suppressed the shoot and root biomass with the increase in salinity. While T2 treatment (saline-alkali solution of 10 g/L) application significantly increased the root activity, soluble sugar, soluble protein, vitamin C and flavonoid content, POD (Peroxidase), AsA (reduced ascorbate), MDA (Malondialdehyde), and reduced the proline content; H_2_O_2_, O_2_, K^+^ content, Na^+^ content, Cl^+^ content, and ratio of Na^+^/K^+^, respectively. Furthermore, plants grown with saline-alkali solution of 30 g/L presented the highest values of proline content, hydrogen peroxide, superoxide radicals and Na^+^ content, Cl^+^ content, ratio of Na^+^/K^+^ with the severity of the saline-alkali solution. The aboveground biomass of ice plant under saline-alkali stress was closely related to the Na^+^, K^+^, Na^+^/K^+^ ratio, proline, SOD, and CAT, indicating that the T2 treatment may alleviate the inhibition of ice plant growth by reducing the Na^+^/K^+^ ratio and improving antioxidant capacity. Therefore, planting ice plant in saline-alkali stress soil can provide additional minerals, phytochemicals, antioxidants, and related nutrients, and it is a vegetable suitable for saline-alkali stress areas.

## Introduction

The ice plant (*Mesembryanthemum crystallinum* L.) has become a model of salt stress response (Ahlawat et al., 2024). Moreover, it can effectively resist salt erosion by improving water use efficiency by converting the C_3_ to CAM photosynthesis mechanism (Atzori et al., 2017, 2020). This annual succulent herbaceous plant is known for its excellent salt tolerance and is a typical representative of halophytes (Naz et al., 2022; Nazir et al., 2023). Soil salinization, alkalinization, and drought are common problems worldwide, which have negative effects on plant development, growth and lead to an imbalance in farming and urban ecologies (Gao et al., 2024; Shi et al., 2024). In arid climate conditions, flat agricultural lands are more susceptible to salinization due to high evapotranspiration (ET) rate and inadequate soil drainage (Libutti et al., 2018; Mukhopadhyay et al., 2021). In addition, Kim et al. (2012) showed that salt reaction leads to the localization of ions, receptors, and organic compounds. As a result, humans consume these bioactive compounds (antioxidants) in large quantities to neutralize health hazards (Kim et al., 2012; Mndi et al., 2023). These stresses can lead to significant reductions (>50%) in total yields of almost all major food crops in worldwide (Kashyap et al., 2017). Soil particles can be divided into two categories: soils rich in salt (NaCl) and soils rich in solute (NaHCO_3_ and Na_2_CO_3_) (Guo et al., 2024). Universally, water scarcity and flood problems strictly limit agricultural productivity (Nasir and Toth, 2022).

Salinity can cause a 50% yield loss (Bagwasi et al., 2020; Muneeba et al., 2024). Food supplies will need to increase by 70% by 2050 to keep pace with population growth, which will put pressure on food supplies (Abbas et al., 2017; Aslam et al., 2023). Salinity stress can cause osmotic stress and ion toxicity, leading to severe growth and yield declines (Shah et al., 2021). Salinity alters cellular ultrastructure, prevents photosynthesis, disrupts membrane structure, produces reactive oxygen species, inhibits enzyme activity, and has other effects on crop development and productivity (Huang et al., 2021; Seleiman et al., 2022). Increasing soil salinization has become a threat to global crop production, and by the end of 2050, more than half of arable land will become saline (Naz et al., 2022; Nazir et al., 2023). Salt-induced osmotic stress reduces water uptake, root and stem cell expansion, stomatal conductance, and carbon dioxide uptake (Alkharabsheh et al., 2021; Hasanuzzaman et al., 2023). In addition, salt stress interferes with enzyme activity, photosynthesis, membrane structure, hormone balance, water and nutrient absorption, and induces oxidative stress (Taha et al., 2021; Ibrahimova et al., 2021). Ice plants are known for their antioxidant properties, which scavenge (ROS) reactive oxygen species. In addition, ice plants contain antioxidant enzymes such as ascorbate peroxidase, SOD, and catalase (Oh et al., 2015). Both temperature and salt concentration initially lead to tissue water stress due to osmotic pressure in the root zone, due to water stress or soluble salt concentration, with widespread negative effects on plant water uptake, nutrient uptake, development, and new root development (Abobatta, 2020). However, as the salt concentration in the soil increases, the plant’s ability to excrete and store salt decreases, resulting in an increase in Na^+^ and Cl in the leaves, leading to salt concentration entering the second most common category of ion-induced toxicity (Tapia et al., 2016). Simultaneously, the levels of ROS were observed to significantly decrease in many plant tissues (Munns and Tester, 2008; Mukhopadhyay et al., 2021; Verslues and Sharma, 2010). But, salt increase in soils can negatively affect plant physiology and anatomy, which can have multiple consequences (Van Zelm et al., 2020). First, the root system is affected by salt stress, causing short- and long-term changes (Ferdosi et al., 2021; Wang et al., 2024). As a direct response to salt, osmotic stress leads to a decrease in water availability, which inhibits plant growth (Asad et al., 2024). Therefore, among the many abiotic stresses affecting global plant productivity, salt is one of the most important issues (Ma et al., 2020). Plants have evolved to synthesize antioxidant (AOX) and antioxidant enzymes that work together to maintain the homeostasis to limit the creation of ROS and the damage they cause (Madhavi et al., 2021; Soares et al., 2019). These metabolites are used to sense oxidative stress, detoxify or neutralize excess ROS, and protect macromolecules from oxidation (Bauchet and Causse, 2012; Miller et al., 2010). The complexity of Na^+^ and Cl^-^ concentrations in plant roots seems to be related to the ability to partition these toxic ions into the vacuole (Mittler, 2017; Mohanta et al., 2018) and to the local concentration and availability of AOX. Studies have shown that the effects of different combinations of stresses on ROS accumulation and antioxidant oxygen (AOX) capacity differ significantly from those observed under single stresses (Müller and Munn´ e-Bosch, 2011; Choudhury et al., 2022). The ability of plants to tolerate drought and salinity is highly dependent on genotype, water availability, and environmental conditions, namely the coexistence of other stress factors (Kong et al., 2013). Good results in terms of stress tolerance have been found in wild relatives of various crops such as ice plant, rice, ice, maize, soybean, cabbage and tomato (Rajagopalan et al., 2016; Barchenger et al., 2019; Quezada-Martinez et al., 2021).

It is noteworthy that ice plant is a facultative halophyte that switches its uptake from C_3_ to CAM photosynthesis in response to excessive salinity, an eco-physiological adaptation that minimizes water loss and helps retain water by opening stomata at night to absorb CO_2_ from outside. During the dark period and early light period of the diurnal cycle, ice plant has a low carbon dioxide compensation point and a significantly lower photorespiration rate compared to C3 plants (Agarie et al., 2009; Cerruti et al., 2024). The morphological parameters depend on the field data, such as the number of leaves and lateral shoots, soil plant analyses development (SPAD), the root and shoot fresh weight and the dry weight of root and shoot (Muhammad et al., 2024). SPAD is important for measuring chlorophyll content in leaves, which mainly determines photosynthesis efficiency and plant development (Muhammad et al., 2024; Li et al., 2024). The underground salt water in southern Xinjiang is large, and the irrigation and drainage water in the field is also salt water. The comprehensive development and utilization of salt water is an urgent problem to be solved. Ice plant is a typical salt-secreting plant. How to use the salt water resources in southern Xinjiang to produce ice plant to determine the utilization threshold, and use the salt absorption characteristics of ice plant to provide a theoretical basis for the improvement of saline-alkali land and the rotation system of vegetable secondary salinization. The objective is to identify the optimal salt concentration that balances plant growth and medicinal value, thereby maximizing the production potential of ice plants in a hydroponic system.

## Materials and methods

### Study site

**Figure 1:**
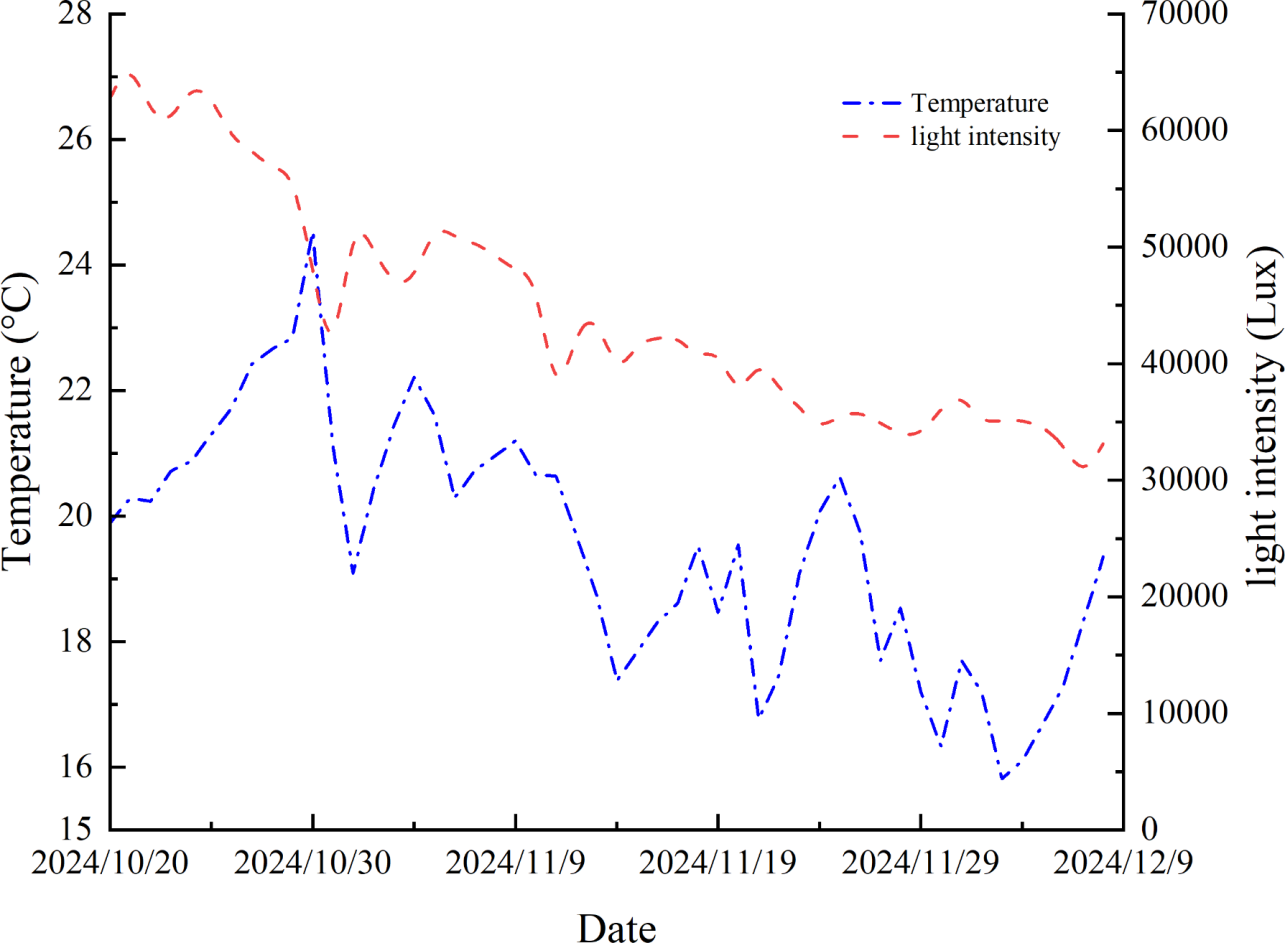
Daily average temperature and light intensity variations of ice plant in greenhouse.

The research was conducted in the solar greenhouse of Awati County, Aksu Prefecture, and Xinjiang Province during October to December 2024. There is a heating device in the greenhouse to confirm the standard growth of ice plant. During the experimental period, daily average temperature and light intensity variations were recorded using the RR-9100 recorder (Beijing Yugen Technology Co. Ltd., Beijing China) as show in **Figure 1**. The experiment selected crystal ice plant as the research object, selected 4 to 5 leaves, and the plants with uniform and strong growth were transplanted into the hydroponic box for 5 days before treatment. The nutrient solution was replaced every 3 to 5 days during the hydroponic process.

### Experimental design

The experiment was conducted with a completely randomized design (CRD) using three replicates. Set 6 concentrations of compound salt stress treatment. During the research, the nutrient solution was exchanged regularly every 3 to 5 days. On the basis of distilled water, irrigation water was made of fresh water and chemicals Na_2_SO_4_, NaCl, NaHCO_3_, CaCl_2_, MgCl_2_ in a mass ratio of 8:8:1:1:1 (simulating the slightly salty water in southern Xinjiang); 100 plants were treated in each treatment, a total of 700 plants, and the ice plant was cultivated for 40 to 50 days. The various physiological indicators were measured when the ice plant was edible. A total of 6 treatments (T1~T6) were set up in the experiment, with the concentrations of the composite saline-alkali solution being 5g/L, 10g/L, 15g/L, 20g/L, 25g/L, and 30g/L, respectively, with pure distilled water as the control (CK). The specific configuration method of the composite saline-alkali solution is to mix Na_2_SO_4_, NaCl, NaHCO_3_, CaCl_2_, MgCl_2_ in the proportion of 8:8:1:1:1 on the basis of distilled water.

### Measurements

#### Growth index

Tags 6 plants for each treatment, and measure their growth indexs every 5 days. Use a tape to measure the plant height in cm. Use a vernier caliper to measure the stem diameter which is 2 cm away from the root zone. Use SPAD-502 instrument to measure the largest leaf. The leaves at the fourth node are measured with a vernier ruler to measure the leaf thickness in (cm). Selected 3 plants of each treatment and measure the above and below dry and fresh weights, root morphology and root activity at the same time, respectively.

#### Physiological indicators

The ice plant was transplanted for 40 to 50 days. The various physiological indicators were measured when the ice plant was edible and reached to the harvest maturity stage. The duration of stress treatment before data measurement was calculated from the number of days after transplantation. Take the same type of tubes, draw 20 micro liters (0.02 ml) of enzyme solution was added 3 ml reaction solution, 4000Lux illumination (multi-annular fluorescent light incubator) 30 minutes (try to be consistent illumination conditions), while taking four test tubes, three do control, a blank (no enzyme solution to buffer instead); blank is set in the dark, the control (CK) and the enzyme solution was placed under the same conditions of illumination 4000Lux 30 minutes, shading save to blank zero, 560nm ratio color. Dark sample is use for machine zero and light sample is use in formula. Total superoxide dismutase (SOD) activity was analysed at 560 nm according to the technique of (Ors et al., 2021). The 20 microliters (0.02 ml) of enzyme solution +3 ml reaction solution and cuvette at 470 nm next time, a total of three times at intervals of one minute to read the readings to the value of absorbance change per minute (ΔA470/min • mg pr or ΔA470/mgFW) indicates the size of the enzyme activity. POD activity was calculated with analysed at 470 nm according to the technique of (Ors and Suarez, 2017). The 0.1 ml sample (or 50 L) +2.5 ml reaction liquid enzyme solution, 240 nm colorimetric, every one minute reading 1, a total of three readings. 2.5 ml reaction liquid is use to make the machine zero. CAT activity was determined according to the technique of (Alam et al., 2021). MDA determination of 1 ml enzyme solution +2 ml of 0.6% of TBA, sealing boiling (100 C^0^) water bath for 15 minutes, then cooled rapidly centrifuged and the supernatant, at 600, 532, 450 nm three wavelengths than the color. 2.5 ml reaction liquid is use to make the machine zero. MDA content was analysed according to the technique of (Sahin et al., 2018). Total soluble protein content was measured by the Coomassie brilliant blue method, 20 micro liters (0.02 ml) of enzyme solution +3 ml G-250 for 2 minutes, 595 Namib color, while the blank (20 microliters phosphate buffer (PH = 7.8): +3 ml G-250) (Bao et al., 2020). Proline content was calculated by the sulfosalicylic acid method, 0.3 g leaf blade, add 5 ml sulfosalicylic acid, stamped, boiling water bath for 10 minutes, filtered, and the filtrate 2 ml draw (at the same time as the blank, draw 2 ml of distilled water), 2 ml of glacial acetic acid and 3 ml of acidic ninhydrin, boiling water bath for 40 minutes, cooled, 5 ml of toluene was added, thoroughly shaken, still stratification, take the upper toluene solution in cuvette, 520 nm colorimetric (Urmi et al., 2023). Total soluble sugar was measured by the anthrone procedure (Zaher-Ara et al., 2016). Quantification of reduced ascorbic acid (AsA) was carried out according to the method of (Gillespie and Ainsworth, 2007) by adding 6% trichloroacetic acid from frozen leaf and evaluating the AsA-mediated reduction of iron ions detected to form complexes with 2, 2-bipyridine at 525 nm. For flavonoid content, the technique defined by Fang et al. (2024) was used. The O^2-^ content was calculated by (Nakano and Asada, 1981) using an amendment of the method. The O^2-^ content at 530 nm was analysed. The H_2_O_2_ content in the supernatant was analysed at 415 nm (Ren et al., 2005; Li et al., 2020).

#### Na^+^ and K^+^ contents

The contents of Na^+^ and K^+^ were measured when the plant was edible and reached the harvest maturity stage. The leaves of ice plant were heat treated for 30 min at 105°C, dried at 75°C and then ground into powder form. Each sample was digested with H_2_SO_4_-H_2_O_2_, and then the Na^+^ and K^+^ contents were determined by using a flame photometer (Ren et al., 2005). At the same time, the nitrogen content was determined using an AA3 flow analyser (Li et al., 2020).

### Statistical analysis

Data analysis is carried out as mean and standard error (SE) were analysed by using two-way analysis of variance (ANOVA) followed by least significant difference (LSD) test at a significance (p ≤ 0.05) level with three replications. Analyses were performed using the STATISTICA application version 13.5.0.17.

## Results and discussion

### Plant growth and morphology parameters

Ice plants grown under different salt treatments showed appreciable differences in morphological parameters such as stem thickness, plant height, number of leaves, leaf area, thick leaves and SPAD to T2 treatment in the presence of saline-alkali solution of 10 g/L are showed in **Table 1**. Halophytes ice plant response to moderate salt concentration promotes their growth, whereas excessive salt concentration impairs their growth (Alkharabsheh et al., 2021; Hasanuzzaman et al., 2023; Eoh et al., 2024). The decrease in root growth under high salt conditions may be due to a shift of energy from growth to maintaining osmotic pressure and facilitating toxin removal processes (Ahmad et al., 2024). Moreover, such contrasts between environmental and salinity stresses reflect plant adaptations, likely to shift energy from shoot growth to help aboveground organisms maintain osmotic balance and improve survival under salinity stresses (Tyśkiewicz et al., 2022). The stem thickness, plant height, number of leaves, leaf area, thick leaves and SPAD were significantly lower under the T6 treatment in the presence of saline-alkali solution of 30 g/L than that the CK treatment and displayed a decreasing trend with increasing stress severity. Drought and salt stress are believed to be important drivers of plant developmental processes that can lead to a decrease in chlorophyll (Fang et al., 2024; Ren et al., 2005). However, treatment of plants with 10 g/l saline led to an increase in these parameters in all combined control treatments. The 10 g/l saline water and the percentage increase in root fresh weight, plant height, leaf number, leaf area, and leaf thicknes were 15.8%, 39.2%, 24.0%, 34.0%, and 31.5%, respectively, than those of 30 g/l saline solution. Kim et al. (2012) also found that ice plant leaf number, lateral leaf number, and leaf area were positively correlated with leaf and leaf biomass. Ahlawat et al. (2024) confirmed that 400 mM NaCl improved the fresh weight of rice seedlings by 2 times compared to the control seedlings. Therefore, high salt concentrations may affect the biomass increase of snow plants.

**Table 1:** The effect of salt treatments on the plant height, stem thickness, number of leaves, leaf area, thick leaves and SPAD value, of ice plants grown under different salt treatments.

Salt treatments	Plant height (cm)	Stem thickness (cm)	Number of leaves	Leaf area (cm^2^)	Thick leaves (cm)	SPAD value
CK	15.7 ± 2.41b	0.93 ± 0.56c	9.3 ± 4.23b	131.0 ± 4.67d	0.21 ± 0.25b	51.8 ± 3.41b
T1	16.7 ± 2.23b	1.06 ± 0.57b	8.3 ± 3.41c	136.0 ± 4.81c	0.24 ± 0.29b	54.3 ± 3.37b
T2	19.0 ± 3.45a	1.19 ± 0.39a	11.7 ± 4.34a	151.9 ± 5.60a	0.28 ± 0.36a	58.3 ± 4.49a
T3	14.3 ± 2.04c	1.14 ± 0.42a	11.3 ± 4.36a	147.7 ± 6.54b	0.28 ± 0.36a	57.1 ± 4.46a
T4	13.5 ± 1.09c	1.05 ± 0.49b	8.7 ± 2.22c	127.2 ± 3.92e	0.21 ± 0.25b	48.6 ± 2.34c
T5	12.4 ± 1.13d	0.96 ± 0.58c	8.3 ± 2.42c	106.7 ± 3.83f	0.17 ± 0.21c	43.6 ± 1.78d
T6	10.1 ± 1.14e	0.89 ± 0.44d	6.3 ± 1.39d	88.7 ± 2.61g	0.17 ± 0.21c	41.2 ± 1.91d

CK: distilled water control; T1: saline-alkali solution of 5 g/L; T2: saline-alkali solution of 10 g/L; T3: saline-alkali solution of 15 g/L; T4: saline-alkali solution of 20 g/L; T5: saline-alkali solution of 25 g/L; T6: saline-alkali solution of 30 g/L. Different letters indicate significant differences between groups (p < 0.05). Values represented means ± SD (n = 9).

**Figure 2:**
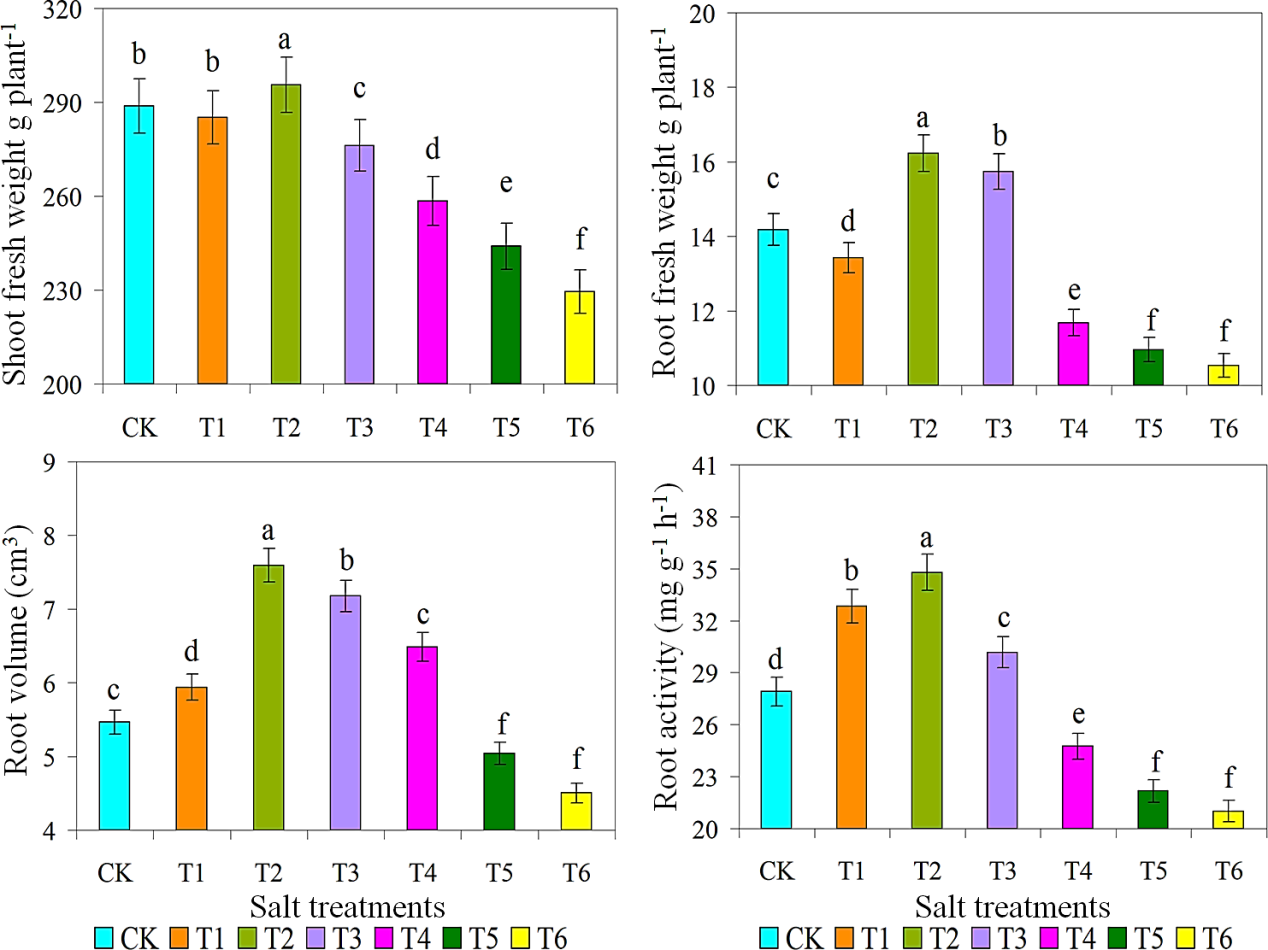
Effect of different salts treatments on shoot fresh weight g plant^-1^, root fresh weight g plant^-1^, root volume and root activity of ice plant. Bar graphs represent the mean ± SE of three replications, and different lowercase letters above the bar graphs indicate significant differences between treatment groups (*p* ≤ 0.05). CK: distilled water control; T1: saline-alkali solution of 5 g/L; T2: saline-alkali solution of 10 g/L; T3: saline-alkali solution of 15 g/L; T4: saline-alkali solution of 20 g/L; T5: saline-alkali solution of 25 g/L; T6: saline-alkali solution of 30 g/L.

Ice plant grown under various salt concentrations indicated a significant variance in properties such as SPAD value, shoot and dry weight (g/plant), root volume, and root activity. The increasing trend of chlorophyll contents observed in 200 mM NaCl solution may indicate that plants are in a stress adaptation stage where they optimize their photosynthetic apparatus to cope with oxidative stress (Bekmirzaev et al., 2020; Wrzecí et al., 2022). Then, as shown in **Table 1**, SPAD values ​​of T2 and T3 treatments were significantly higher, while SPAD values ​​of T5 and T6 treatments were lower. Moreover, this opposite behaviour between moisture and salt stresses reflects a plant adaptation, which may be shifting energy from root growth to helping roots osmotic pressure and improve survival at moderate salt stress (Quezada-Martinez et al., 2021). Root weights were measured to investigate their growth under diverse saline and alkaline conditions. When grown in 10 g/L saline-alkaline solution, shoot and root FW of plant 1 were 299 and 17 g/plant, respectively (**Figure 2**). Similarly, root volume and root activity of T2 treatment were considerably greater than all other treatments, respectively. Bohnert and Cushman (2000) reported that accumulate salt storing large amounts of NaCl and CaCl_2_ in the shoot. Furthermore, this salt-loving plant grows well in a salt environment with salinity ranging from 100 to 400 mM (Asad et al., 2024).

### Total soluble sugar, protein, vitamin C and flavonoid content of ice plant

**Figure 3:**
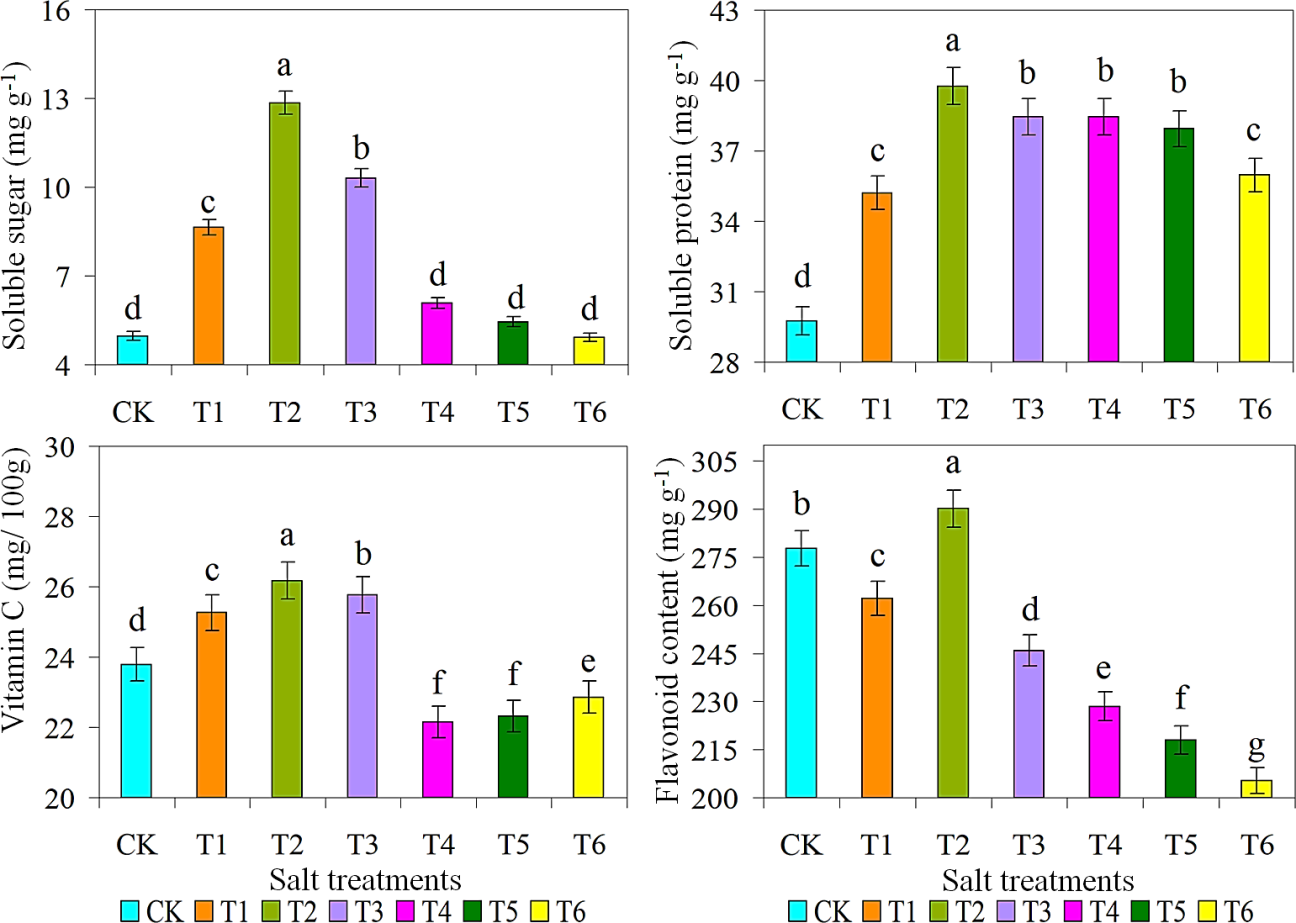
Effect of different salts treatments on soluble sugar, soluble protein, vitamin C and flavonoid content of ice plant. Bar graphs represent the mean ± SE of three replications, and different lowercase letters above the bar graphs indicate significant differences between treatment groups (*p* ≤ 0.05). CK: distilled water control; T1: saline-alkali solution of 5 g/L; T2: saline-alkali solution of 10 g/L; T3: saline-alkali solution of 15 g/L; T4: saline-alkali solution of 20 g/L; T5: saline-alkali solution of 25 g/L; T6: saline-alkali solution of 30 g/L.

Exogenous application of saline-alkaline solution significantly affected the soluble protein, total sugar, vitamin C and flavonoid of ice plant under the salt stress condition (**Figure 3**). Compared with CK, different degrees of saline-alkaline solution salt stress significantly improved the soluble protein, total sugar, vitamin C and flavonoid of T2 treatment, which were 12.9, 40.1 and 290.1 ​​mg g^-1^, respectively. The vitamin C content was 26.2 mg/100g, which was higher than all other treatments. Therefore, MT application could increase the proline of plants under saline soil, which could help increase the osmotic potential of leaves, enhance water absorption, promote photosynthesis, and ultimately improve the characteristics of plants under saline soil (Zaher-Ara et al., 2016). The effect of saline-alkaline solution application depends on the type of application and the concentration of the saline-alkaline solution. The contents of soluble total sugar, protein, vitamin C, and flavonoid compounds in both T1 and T2 treatments first increased with the increase of saline-alkaline solution concentration, and all reached a peak value of 10 g L^-1^ in T2 treatment, and then decreased with further increase of saline-alkaline solution concentration. Flavonoids content can protected from biotic and abiotic stresses through antioxidant activities (Taha et al., 2021; Ibrahimova et al., 2021). In T6 treatment, the soluble total sugar and flavonoid contents monotonically reduced with the intensification of saline-alkaline solution concentration and reached the lowest value. The soluble total protein content of 10 g L^-1^ saline-alkaline solution under salt stress increased significantly compared with CK treatment, and the application of saline-alkaline solution had a significant effect on the soluble total protein content in T2 and T3 treatments. Under CK treatment, the soluble protein and sugar content decreased considerably, and the largest increase occurred at 10 g L^-1^. Under T4 and T5 treatments, the application of saline-alkaline solution significantly decreased vitamin C, and the smallest decrease occurred at 20 and 25 g L^-1^, respectively. Under salt stress conditions, plants accumulate water-retaining compounds, soluble sugars (Muhammad et al., 2024), and proline (Li et al., 2024) to maintain water balance and prevent plant dehydration under drought conditions. Proline helps scavenge ROS, stabilize cell membranes, and inhibit protein and enzyme degradation under salt stress (Ors et al., 2021). **Table 2** shows the Pearson correlation coefficients between antioxidant enzyme activities, reactive oxygen species, antioxidants, growth factors, protein, proline, and MDA contents of snow leaf leaves. A significant positive correlation was observed between flavonoid content and H_2_O_2_ content, K^+^, LA, plant height, SPAD, dry weight, and dry weight, whereas a negative correlation was observed with protein content.

**Table 2:** Pearson’s correlation coefficients of antioxidant enzyme activities, reactive oxygen species (ROS), protein, proline and MDA contents of ice plant.

	AsA	CAT	Cl^+^	FC	H_2_O_2_	K^+^	LA	MDA	Na^+^	Na^+^/K^+^	O_2_	POD	PH	SOD	SPAD	SW	SP	PC
CAT	0.44																	
Cl^+^	0.43	0.30																
FC	0.09	0.04	-0.72*															
H_2_O_2_	-0.27	-0.06	0.53*	-0.74*														
K^+^	-0.24	-0.12	-0.96**	0.86**	-0.69*													
LA	0.37	0.56*	-0.37	0.82**	-0.74*	0.60*												
MDA	0.58*	0.79**	0.53*	0.04	0.12	-0.35	0.42											
Na^+^	0.28	0.22	0.98**	-0.78*	0.61*	-0.97**	-0.47	0.44										
Na^+^/K^+^	0.06	-0.12	0.82*	-0.91**	0.84**	-0.94**	-0.82**	0.11	0.87**									
O_2_	-0.04	-0.20	0.77*	-0.90**	0.84**	-0.90**	-0.84**	0.01	0.83**	0.98**								
POD	0.44	0.83**	0.37	0.18	-0.17	-0.15	0.61*	0.89**	0.29	-0.12	-0.23							
PH	0.28	0.11	-0.54*	0.95**	-0.84**	0.73*	0.87**	0.14	-0.62*	-0.86**	-0.86**	0.32						
SOD	0.02	0.65*	0.41	-0.46	0.08	-0.35	0.04	0.23	0.42	0.19	0.16	0.41	-0.37					
SPAD	0.45	0.52*	-0.40	0.84**	-0.73*	0.63*	0.98**	0.39	-0.51	-0.82**	-0.84**	0.52	0.87**	-0.05				
SW	0.17	0.14	-0.75*	0.91**	-0.87**	0.89**	0.84**	-0.07	-0.82**	-0.97**	-0.96**	0.12	0.88**	-0.22	0.87**			
SP	0.68*	0.49	0.82**	-0.22	0.07	-0.65	0.16	0.76*	0.75*	0.39	0.32	0.70*	0.01	0.29	0.11	-0.29		
PC	-0.1	0.15	0.74*	-0.78*	0.52	-0.78*	-0.49	0.19	0.81**	0.72*	0.68*	0.25	-0.66*	0.62*	-0.63*	-0.79*	0.46	
RW	0.39	0.50*	-0.42	0.85**	-0.55*	0.61*	0.91**	0.47	-0.52	-0.76*	-0.79*	0.51	0.82**	-0.20	0.95**	0.80**	0.06	-0.67*

AsA, reduced ascorbate; CAT, Catalase; Cl^+^ content; FC, flavonoid content; H_2_O_2_, hydrogen peroxide; K^+^ content; LA, leaf area; MDA, Malondialdehyde; Na+ content, ratio of Na^+^/K^+^; O_2_, superoxide radicals; POD, Peroxidase; PH, plant height; SOD, Superoxide dismutase; SW, shoot weight; RW, root weight; SP, soluble protein; PC, proline content.

Significant at the 0.01 (**) probability level; Significant at the 0.05 (*) probability level.

### Antioxidant and toxic compound in ice plant

**Figure 4:**
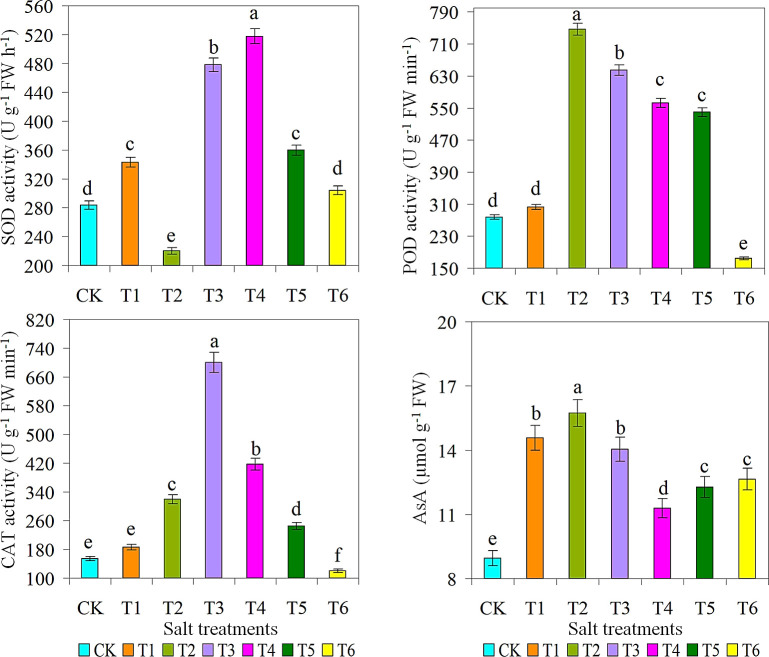
Effect of different salts treatments on SOD, Superoxide dismutase; POD, Peroxidase; CAT, Catalase; AsA, reduced ascorbate of ice plant. Bar graphs represent the mean ± SE of three replications, and different lowercase letters above the bar graphs indicate significant differences between treatment groups (*p* ≤ 0.05). CK: distilled water control; T1: saline-alkali solution of 5 g/L; T2: saline-alkali solution of 10 g/L; T3: saline-alkali solution of 15 g/L; T4: saline-alkali solution of 20 g/L; T5: saline-alkali solution of 25 g/L; T6: saline-alkali solution of 30 g/L.

The SOD, POD, CAT, and AsA activities were considerably different among the different water treatments (**Figure 4**). These factors are essential for reducing ROS-induced damage by increasing the plant’s disease resistance, especially in the root system, which is consistent with the decrease observed in vascular plants (Muneeba et al., 2024; Fang et al., 2024). The SOD and CAT were significantly higher in T3 treatment compared to all other treatments. Whereas, the activities of POD and AsA were significantly higher in T2 treatment than in other treatments. However, there were also significant differences in the formation of toxins among the different aqueous solutions, and the patterns of antioxidant enzymes also differed. The 500 mM NaCl showed the ability to form a protective layer and increase the weight of the cells. Plant flavonoids have free radical scavenging and free radical scavenging effects, which may involve the following mechanisms: (a) inhibition of ROS production by inhibition or activation of free radical scavengers; (b) ROS scavenging; and (c) increased immunity (Ahmad et al., 2024; Hussain et al., 2024). The T2 treatment (10 g L^-1^) produced the highest MDA content compared with all other treatments (**Figure 4**). Application of 30 g L^-1^ saline solution under saline conditions had a significant effect on ROS production in ice plant. Furthermore, significant positive correlations were observed between stem dry weight and CAT, FC, K^+^, LA, plant height, SPAD, and surface roughness. Correlation analysis also showed positive correlations between MDA and POD, soluble protein, AsA, CAT, and Cl^+^ levels.

**Figure 5:**
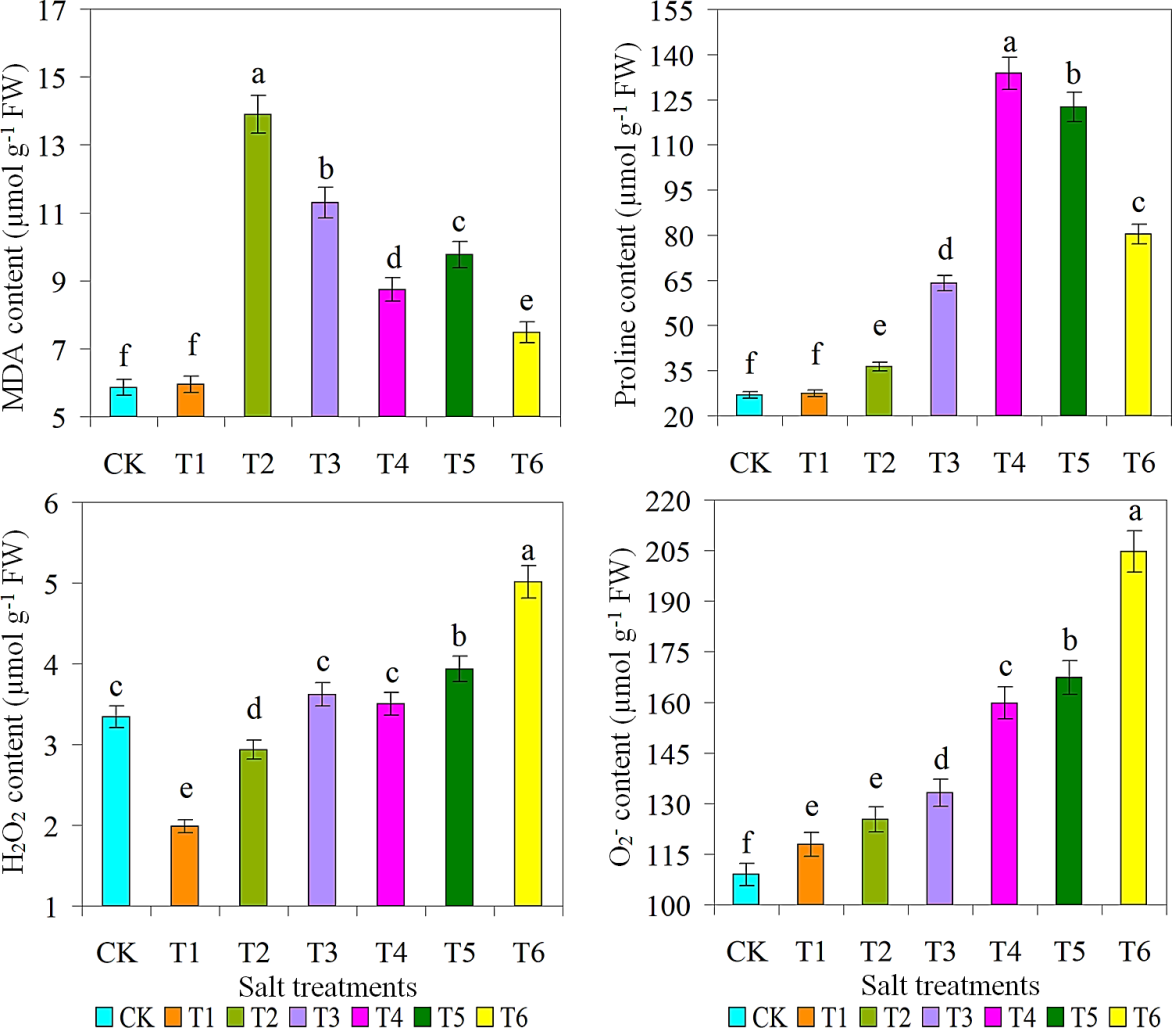
Effect of different salts treatments on MDA, Malondialdehyde; proline content; H_2_O_2_, hydrogen peroxide; O_2_, superoxide radicals of ice plant. Bar graphs represent the mean ± SE of three replications, and different lowercase letters above the bar graphs indicate significant differences between treatment groups (*p* ≤ 0.05). CK: distilled water control; T1: saline-alkali solution of 5 g/L; T2: saline-alkali solution of 10 g/L; T3: saline-alkali solution of 15 g/L; T4: saline-alkali solution of 20 g/L; T5: saline-alkali solution of 25 g/L; T6: saline-alkali solution of 30 g/L.

The highest SOD content and production were observed under the condition of 15 g L^-1^ saline solution. This is because these ROS play a critical role in key signaling pathways associated with the stress response (Alam et al., 2021; Urmi et al., 2023; Fang et al., 2024). Inhibition of these types of ROS and their subsequent damage by SOD and CAT is a key mechanism of action (Madhavi et al., 2021; Aslam et al., 2023). As expected, the CK treatment had the lowest levels of CAT, POD, and AsA. However, there was non-significant variance in proline content between the CK and T1 treatments, with T1 treatment producing significantly lower levels of proline. The production of ROS (H_2_O_2_ and O_2_^-^) was the highest when 30 g L-1 of saline solution was applied, while the production of ROS (H_2_O_2_ and O_2_^-^) was the lowest in the T1 treatment with 5 g L^-1^ of saline solution (**Figure 5**). Plants use a variety of antioxidants to regulate ROS production and thus prevent salt stress-induced damage by effectively scavenging ROS or restoring intracellular antioxidant levels (Seleiman et al., 2022; Hussain et al., 2024). There was no difference in H_2_O_2_ production between T3 and T4 treatments. However, the highest POD, AsA, and MDA activity values ​​were detected in the T2 treatment. While on other hand, the highest levels of SOD and proline were present in T4 treatment. It is known that abiotic factors such as salinity and drought stresses increase the ROS activity, which induces oxidative stress and plant oxidative pathways (Tyśkiewicz et al., 2022; Nazir et al., 2023). The strong interaction network emphasized the important role of polyphenols in the antioxidant activity of *M. crystallinum* under salt stress, indicating that the pathogen system was more efficient under optimal salt stress conditions (Bekmirzaev et al., 2020; Aslam et al., 2023; Ahmad et al., 2024).

### Ion contents in ice plant

**Figure 6:**
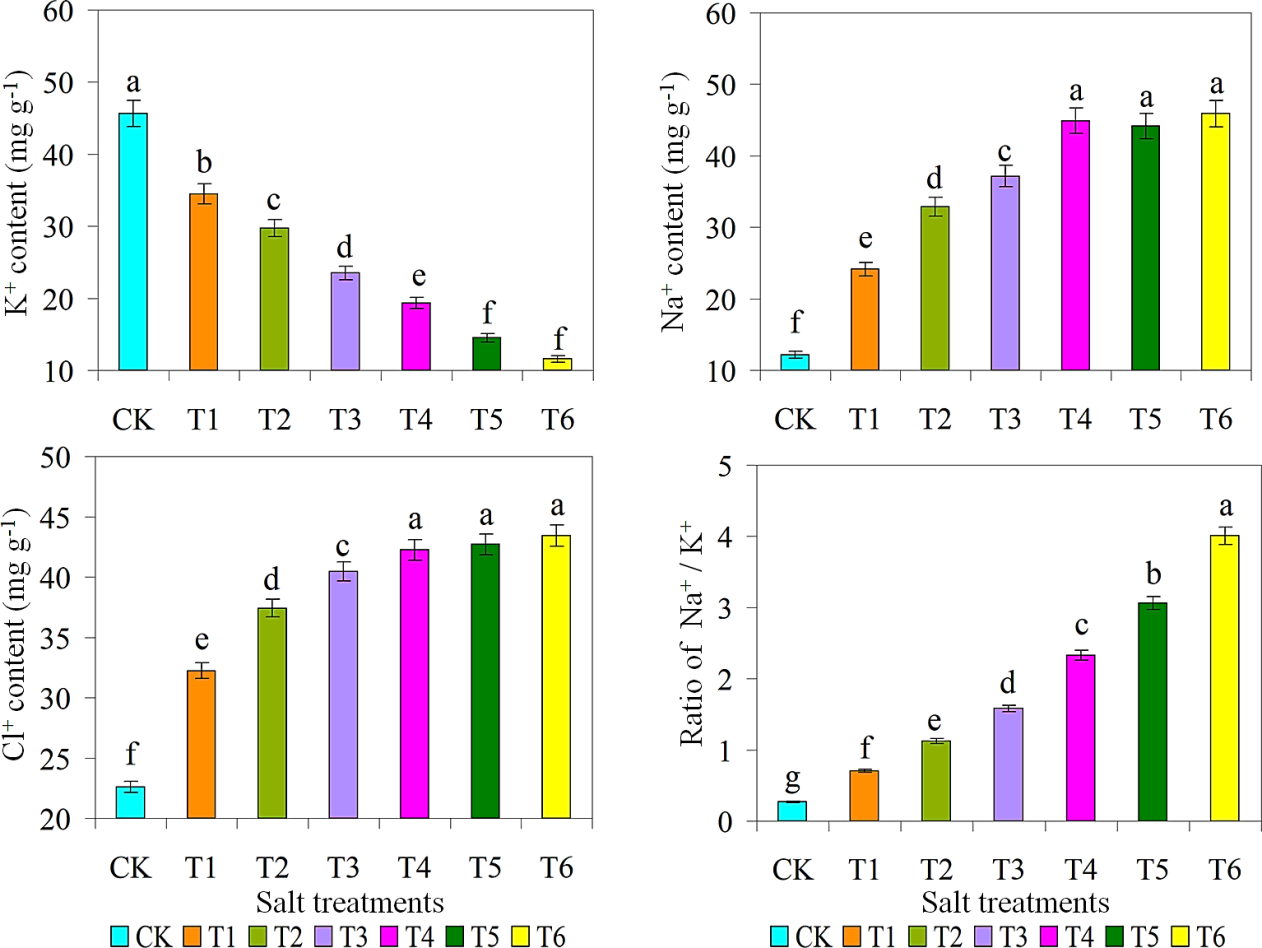
Effect of different salts treatments on K^+^ content, Na^+^ content, Cl^+^ content, ratio of Na^+^/K^+^ of ice plant. Bar graphs represent the mean ± SE of three replications, and different lowercase letters above the bar graphs indicate significant differences between treatment groups (*p* ≤ 0.05). CK: distilled water control; T1: saline-alkali solution of 5 g/L; T2: saline-alkali solution of 10 g/L; T3: saline-alkali solution of 15 g/L; T4: saline-alkali solution of 20 g/L; T5: saline-alkali solution of 25 g/L; T6: saline-alkali solution of 30 g/L.

The results showed that snow plant accumulated more K^+^ under CK treatment, while the contents of Na^+^, Cl^+^, and Na^+^/K^+^ were higher under T6 treatment (**Figure 6**). Na^+^ is the major ion responsible for salt degradation in plants. Since the ionic radius and adsorption capacity of Na^+^ are comparable to those of K^+^, higher concentrations of Na^+^ and decrease K^+^ absorption by inhibiting movement, resulting in Na^+^ toxicity and K^+^ leaching (Shah et al., 2021; Huang et al., 2021). There was no significant difference in Na^+^ retention in the leaves of snow-covered plants under T4, T5, and T6 treatments. In addition, the Na^+^ content in T4, T5, and T6 treatments was highest because salt transport from root cells to the mesophyll depends on inositol, and inositol interactions facilitate the uptake and long-distance transport of salt. It has been reported that many halophytes accumulate Na^+^ in their leaves and roots in biosaline cultivation, which leads to a decrease in cation such as K^+^ (Ors et al., 2021; Bagwasi et al., 2020). According to the results of K^+^ ion determination, CK application had the highest value, and T6 application had the lowest value. There was non-significant variance in K^+^ ion accumulation between T5 and T6 applications. These results indicate that lower CaCl_2_ concentrations affect K^+^ ion accumulation in leaf tissues. However, Na^+^, and K^+^, sorption rates were higher in CaCl_2_ salt application because NaCl inhibits K^+^ ion concentration, while CaCl_2_ increases Cl and Ca^2+^ content in leaves, leading to the sorption of other cations (Aslam et al., 2023; Hasanuzzaman et al., 2023). As Cl^+^ levels increased, significant differences were observed in the T4 (41 ± 20 g/L) and T5 (43 ± 25 g/L) treatments.

The excretion, transport, and sequestration of toxic ions are highly relevant to water conservation, which will be discussed later, as efficient water conservation requires a balance between Na^+^ and Cl^+^ in the vacuole and between K^+^ and solute receptors. The Na^+^ concentration and Na^+^/K^+^ ratio of the T1 was considerably lower as compare with other treatments, and the other treatments were relatively equal. The Na^+^/K^+^ ratio in the T6 were considerably higher as compared with CK treatment. The Na^+^/K^+^ ratio was the lowest in CK and T1 treatments. On the other hand, the T6 treatment (15 g of/L) had the maximum Na^+^, Cl^+^, and Na^+^/K^+^ ratios. K^+^ and Na^+^ have similar chemical properties, so their absorption and distribution are based on a common transport system, and excess Na^+^ can compete with K^+^. This affects the Na^+^/K^+^ ratio and leads to K^+^ deficiency, which in turn affects growth and stress resistance (Ren et al., 2005; Abbas et al., 2017; Huang et al., 2021). There are many Limitations of hydroponics studies over conventional agriculture, there are some, higher set up cost, growers require skill and knowledge to maintain optimum production in commercial applications (Nazir et al., 2023), because each plant in a hydroponics system is sharing the exact same nutrient, diseases and pests can easily affect each plant, plants react quicker to changes in the environment (Muneeba et al., 2024), however, if this change is for the worst, plants will quickly react to it; showing signs of deficiency or trouble, hot weather and limited oxygenation may limit production and can result in loss of crops (Hasanuzzaman et al., 2023).

## Conclusion

Exploring the stress resistance mechanism of the ice plant is becoming a hot research direction for plant physiologists. This study indicated that saline-alkali stress soil affected the production of phytochemicals, food growth, and nutritional value in ice plant leaves. This study indicated that with the increase of salinity, T6 (30 g/L saline-alkali solution) considerably decreased the stem diameter, plant height, SPAD, and leaf area of ice plant and significantly inhibited the biomass of aboveground and roots. T2 treatment (10 g/L saline-alkali solution) significantly increased root activity, soluble sugar, soluble protein, vitamin C and flavonoid content, POD, AsA, MDA, and significantly decreased the proline content; H_2_O_2_, O_2_^-^, K^+^, Na^+^, Cl^+^ and Na^+^/K^+^ ratio, respectively. In addition, plants grown in 30 g/L saline-alkali solution showed the highest proline content, hydrogen peroxide, superoxide radicals. This indicates that T2 application can alleviate the inhibition of ice plant growth by biomass in saline-alkali land by reducing the Na^+^/K^+^ ratio and improving antioxidant defence system. These findings provide a deeper understanding of ice plants to reflect the degree of tolerance to combined saline-alkali salt stress soil to provide a theoretical basis for the sustainable development of the ice plants industry in arid and saline-alkali salt stress areas. Promoting the cultivation of this highly nutritional plant in moderately saline environments may thus present a specific interest. In addition, further studies seem to be required to clarify the effect of different types of salt treatments on the growth and secondary metabolism of ice plants.

## Data Availability

The original contributions presented in the study are included in the article/**Supplementary Material**. Further inquiries can be directed to the corresponding author.
